# The hidden cost of patient advocacy: moral courage mediating clinical advocacy behaviors and workplace ostracism among Chinese nurses

**DOI:** 10.3389/fpsyg.2026.1835394

**Published:** 2026-06-01

**Authors:** Li Shang, Na Wang, Liping Qin, Ling Song, Suyao Yan, Jin Zhang

**Affiliations:** 1 Department of Pediatric Emergency and PICU, Northwest Women’s and Children’s Hospital, Xi’an, China; 2 Department of Pediatrics III, Northwest Women’s and Children’s Hospital, Xi’an, China

**Keywords:** moral courage, nursing management, patient advocacy, perceived organizational support, workplace ostracism

## Abstract

**Background:**

Patient advocacy is a central ethical responsibility of nurses and is generally associated with patient protection, safety, and care quality. However, most existing studies have emphasized the benefits of advocacy for patients, whereas relatively little is known about whether advocacy may create interpersonal costs for nurses themselves. This issue is particularly relevant in Chinese tertiary hospital settings, where nurses work within high-demand, team-based, and hierarchical clinical environments. Yet, empirical evidence on how clinical advocacy behaviors are associated with workplace ostracism, and through which psychological and organizational mechanisms this association may occur, remains limited.

**Methods:**

This study investigated the relationship between clinical advocacy behaviors (CAB) and workplace ostracism (WO) among Chinese nurses, and further examined the mediating role of moral courage (MC) and the moderating role of perceived organizational support (POS). A cross-sectional study was conducted with 350 nurses from tertiary hospitals in Xi’an, China. Participants completed validated measures of clinical advocacy behaviors, moral courage, workplace ostracism, and perceived organizational support. Internal consistency was acceptable to excellent for all instruments (Cronbach’s *α* = 0.718–0.919). Data were analyzed using hierarchical regression, bootstrap-based mediation analysis (5,000 resamples), and subgroup analyses.

**Results:**

CAB was significantly and positively associated with WO (*β* = 0.329, *t* = 7.194, *p* < 0.001). MC partially mediated this relationship, with an indirect effect of 0.105 (95% bootstrap CI: 0.027 to 0.188), representing 32.0% of the total effect. Significant heterogeneity was identified across departments and professional titles, with the strongest CAB–WO associations found among entry-level nurses (*r* = 0.602, *p* < 0.001) and emergency department nurses (*r* = 0.428, *p* < 0.001). The moderating effect of POS was not statistically significant.

**Conclusion:**

Nurses’ clinical advocacy behaviors are associated with greater workplace ostracism, suggesting that patient advocacy may carry interpersonal costs in clinical environments. Moral courage serves as a partial explanatory mechanism, whereas perceived organizational support does not significantly attenuate this relationship. Interventions are needed to foster organizational climates in which nurses can advocate for patients without incurring social exclusion.

## 1 Introduction

### 1.1 Research background

Patient advocacy is a core ethical and professional responsibility in nursing, referring to actions undertaken to protect patients’ rights, facilitate informed decision-making, prevent harm, and promote equitable access to care (Bu and Jezewski, 2007). Because nurses maintain the most continuous contact with patients, they are often in a privileged position to recognize unmet needs, identify threats to patient welfare, and intervene on behalf of vulnerable individuals. Advocacy is therefore widely regarded as a hallmark of professional nursing practice. Consistent with this view, the American Nurses Association identifies advocacy as a central nursing obligation, and prior studies have shown that advocacy contributes to improved patient outcomes, enhanced care quality, and stronger therapeutic nurse–patient relationships (Hanks, 2008; American Nurses Association, 2025; Vaartio et al., 2006; Davoodvand et al., 2016).

However, advocacy may also entail interpersonal and professional risks. In many clinical contexts, advocating for patients requires nurses to question treatment decisions, challenge established routines, or voice concerns that may conflict with the preferences of physicians, managers, or coworkers. Although such actions are ethically justified, they may be interpreted as disruptive, confrontational, or inconsistent with prevailing organizational norms. As a result, advocacy may expose nurses to subtle social sanctions and strained workplace relationships (Thacker, 2008; American Nurses Association, 2022). Compared with the substantial literature documenting the benefits of advocacy for patients, far less is known about its possible social costs for nurses themselves.

One potential consequence is workplace ostracism, defined as the perception of being ignored, excluded, or rejected by others at work (Robinson et al., 2013). Workplace ostracism differs from overt mistreatment in that it is often enacted through subtle, ambiguous, and low-visibility behaviors, making it difficult to identify and address through formal channels (Williams, 2007). Nevertheless, its consequences can be substantial. In nursing populations, ostracism has been associated with employee silence, emotional exhaustion, unethical behavior, and other adverse work-related outcomes (Qi et al., 2020). Because nursing work relies heavily on communication, coordination, and team integration, exclusion from workplace relationships may be particularly detrimental in clinical environments.

Chinese nursing settings provide an important context for examining this issue. China has a large and rapidly developing nursing workforce, with 5.63 million registered nurses by the end of 2023 and approximately four registered nurses per 1,000 population (National Health Commission of the People’s Republic of China, 2024). At the same time, nurses in tertiary hospitals often work under conditions of high patient volume, intensive interprofessional coordination, and strong role-based hierarchies. Empirical studies have also documented substantial interpersonal strain in Chinese healthcare workplaces. For example, prior research among Chinese nurses has shown that workplace ostracism is associated with emotional exhaustion and unethical behavior, and recent evidence indicates that workplace psychological violence and verbal aggression remain salient occupational risks for Chinese nurses (Li et al., 2024). These findings suggest that the Chinese nursing workplace is not only clinically demanding but also relationally complex. In such settings, advocacy behaviors that require questioning decisions, raising concerns, or challenging established routines may be especially likely to generate social tension or exclusionary responses.

This issue may be especially salient in China. The Chinese healthcare workplace is shaped in part by cultural norms emphasizing harmony, face-saving, and hierarchical deference, which may render open disagreement or upward challenge socially sensitive even when undertaken in patients’ best interests (Hwang, 1987). At the same time, high service demand, workforce strain, and systemic pressures within the healthcare system may further reduce tolerance for behaviors perceived as interrupting workflow or generating interpersonal tension (Yip and Hsiao, 2008). Within such a context, nurses who engage in advocacy may face an elevated risk of social exclusion.

Despite these considerations, research directly examining the relationship between clinical advocacy behaviors and workplace ostracism remains scarce, particularly in Chinese nursing settings. Most existing studies have examined advocacy as an ethical ideal or ostracism as a general workplace stressor, with limited attention to how the former may contribute to the latter. Addressing this gap is essential for understanding the hidden interpersonal costs of ethically grounded nursing practice and for developing organizational environments in which patient advocacy can be sustained without penalizing those who perform it.

### 1.2 Theoretical foundation

The association between clinical advocacy behaviors (CAB) and workplace ostracism (WO) can be explained by two complementary theoretical perspectives: Conservation of Resources (COR) theory and Social Identity Theory. COR theory proposes that individuals strive to acquire, retain, and protect valued resources, including social acceptance, collegial support, professional reputation, and psychological security at work (Hobfoll, 1989). From this perspective, CAB may be ethically justified and professionally desirable, but it may also consume or threaten nurses’ relational resources. When nurses advocate for patients by questioning treatment decisions, raising safety concerns, or challenging established routines, coworkers or supervisors may perceive these behaviors as disruptive to workflow, hierarchy, or interpersonal harmony. As a result, nurses who engage in advocacy may experience resource loss in the form of weakened peer support, strained professional relationships, reduced informal communication, or social distancing. These resource losses provide a theoretical explanation for why CAB may be positively associated with WO.

Social Identity Theory offers a complementary explanation by focusing on group membership, group norms, and perceived norm deviation. This theory suggests that individuals derive part of their self-concept from group membership and tend to maintain behaviors that preserve in-group cohesion and normative consistency (Tajfel and Turner, 1979). In clinical teams, nurses are often embedded in departmental norms, role expectations, authority gradients, and implicit rules regarding coordination and deference. When advocacy requires nurses to question decisions, challenge routines, or express disagreement, such behavior may be interpreted by others as a deviation from expected group conduct. Empirical work on workplace exclusion also suggests that perceived interpersonal or behavioral deviation can contribute to exclusionary responses in organizational settings (Hitlan and Noel, 2009). Therefore, WO may emerge not only as a reaction to interpersonal disagreement, but also as a subtle social regulatory response toward nurses who are perceived as disrupting group norms or role boundaries.

Together, COR theory and Social Identity Theory provide the theoretical basis for the direct relationship between CAB and WO. COR theory explains why advocacy may threaten nurses’ valued interpersonal resources, whereas Social Identity Theory explains why advocacy may be interpreted as norm-deviating behavior that elicits exclusionary responses. These perspectives support the expectation that nurses who engage more frequently in clinical advocacy may be more likely to experience workplace ostracism.

### 1.3 Research mechanisms

Although COR theory and Social Identity Theory explain why CAB may be associated with WO, additional mechanisms are needed to clarify how and under what conditions this association may unfold. In the present study, moral courage (MC) is conceptualized as an individual-level mediating mechanism, whereas perceived organizational support (POS) is conceptualized as an organizational-level moderating mechanism.

MC refers to the willingness and capacity to act in accordance with ethical principles despite fear, risk, opposition, or anticipated adverse consequences. In nursing, MC is particularly relevant because patient advocacy often requires nurses to speak up in morally sensitive situations, persist in ethically appropriate action, and tolerate the possibility of interpersonal or professional cost (Lachman, 2007). Broader organizational ethics research has conceptualized professional moral courage as a multidimensional capacity involving principled action under threat, endurance in the face of opposition, and commitment to ethical goals (Sekerka et al., 2009). More recent healthcare research has further operationalized MC and demonstrated its relevance to patient safety-related speaking up and ethically challenging clinical action (Martinez et al., 2016).

In this study, MC is expected to mediate the association between CAB and WO. Nurses who engage more actively in clinical advocacy are likely to report higher levels of MC because advocacy often requires ethical conviction, persistence, and willingness to accept personal risk. At the same time, morally courageous nurses may become more visible as individuals who challenge unsafe practices, question decisions, or resist inappropriate routines. Such visibility may increase the likelihood that coworkers or supervisors perceive them as disruptive, confrontational, or inconsistent with prevailing team expectations. Therefore, CAB may be associated with greater WO partly because advocacy is linked to morally courageous action, which can make nurses more vulnerable to social exclusion.

POS refers to employees’ general belief that the organization values their contributions and cares about their well-being (Eisenberger et al., 1986). Organizational support theory suggests that when employees perceive strong support from their organization, they are more likely to view valued work behaviors as legitimate, protected, and recognized by the organization (Rhoades and Eisenberger, 2002). In the context of clinical advocacy, high POS may signal to nurses that patient-protective behaviors are institutionally endorsed rather than personally risky. Under such conditions, nurses may feel more secure when raising concerns, challenging unsafe practices, or speaking up for patients.

Accordingly, POS is expected to function as a contextual boundary condition in the CAB-WO relationship. When POS is high, nurses who engage in advocacy may be less likely to experience exclusion because their behavior is perceived as aligned with organizational values and supported by formal structures. Conversely, when POS is low, nurses may perceive that advocacy lacks organizational backing, and coworkers’ or supervisors’ exclusionary responses may become more consequential. Therefore, the positive association between CAB and WO is expected to be weaker when POS is high and stronger when POS is low.

Taken together, the theoretical foundation and research mechanisms provide an integrated rationale for the proposed model. COR theory and Social Identity Theory explain why CAB may increase nurses’ risk of WO. MC specifies an individual-level pathway through which advocacy may translate into interpersonal cost. POS specifies an organizational boundary condition that may shape the strength of this relationship. This structure clarifies that MC and POS are not theoretical foundations themselves, but mechanism variables linking CAB to WO.

### 1.4 Literature review

Patient advocacy has long been regarded as a core element of professional nursing identity. In nursing practice, advocacy refers to nurses’ actions to protect patients’ rights, safeguard patient welfare, support informed decision-making, prevent harm, and promote access to appropriate care. Early conceptual work emphasized that advocacy is not merely an interpersonal helping behavior, but a professional responsibility embedded in nurses’ ethical commitment to patients (Mallik, 1997). Empirical accounts further showed that nurses may enact advocacy both reactively, by responding to immediate threats to patient interests, and proactively, by anticipating risks and initiating protective actions before harm occurs (Snowball, 1996). These studies established patient advocacy as a central component of nursing ethics and professional practice.

The development of the Protective Nursing Advocacy Scale further advanced this field by enabling patient advocacy to be measured systematically in quantitative research. The scale operationalized protective nursing advocacy as a multidimensional construct involving advocate actions, work context and consequences, environmental and educational influences, and support and barriers to advocacy (Hanks, 2010). Subsequent cross-cultural validation work extended the applicability of the scale beyond its original context and confirmed that advocacy-related beliefs and behaviors could be assessed in different cultural and healthcare systems (Tomaschewski-Barlem et al., 2015). However, most research using this measurement tradition has focused on the level, structure, correlates, and professional value of advocacy. Relatively little attention has been paid to whether clinical advocacy behaviors may generate negative interpersonal responses toward nurses themselves, particularly when advocacy involves questioning decisions, challenging routines, or raising concerns that others may perceive as disruptive.

Workplace ostracism provides a useful construct for understanding such possible interpersonal consequences. Workplace ostracism refers to employees’ perception that they are ignored, excluded, or socially rejected by others at work. Unlike overt aggression or bullying, ostracism is often subtle and ambiguous, occurring through avoidance, silence, withholding information, or exclusion from informal interactions. Prior research has shown that workplace ostracism can lead to psychological distress and maladaptive coping responses (Wu et al., 2012). It has also been linked to dysfunctional workplace conduct, such as knowledge hiding (Zhao et al., 2016), and to changes in deviant and helping behaviors that can undermine interpersonal functioning and organizational effectiveness (Peng and Zeng, 2017). Although this literature has clarified many harmful outcomes of ostracism, it has generally treated ostracism as an antecedent of employee attitudes and behaviors. Less is known about ethically meaningful behaviors that may trigger ostracism. In nursing settings, this limitation is especially important because clinical advocacy may require nurses to voice disagreement, challenge authority gradients, or disrupt routine coordination in order to protect patients.

Moral courage is another important construct for understanding the interpersonal cost of advocacy. Moral courage refers to the willingness and capacity to act according to moral principles despite fear, uncertainty, opposition, or possible personal cost. In nursing, moral courage is particularly relevant when nurses must speak up about unsafe care, defend patients’ rights, or persist in ethically appropriate action despite potential criticism. Conceptual analysis has identified moral courage as a distinct ethical capacity involving moral agency, risk acceptance, perseverance, and commitment to doing what is right (Numminen et al., 2017). Recent empirical research among Chinese nurses has further shown that moral courage is a measurable and clinically relevant professional attribute shaped by individual and organizational factors (Hu et al., 2022). Nevertheless, existing studies have largely framed moral courage as a desirable ethical quality or as a facilitator of professional integrity. Its potential role as a mechanism linking clinical advocacy behaviors to workplace ostracism remains insufficiently examined. This is a notable gap because morally courageous nurses may be more willing to engage in visible advocacy, but such visibility may also make them more vulnerable to being perceived as challenging group norms or authority structures.

Perceived organizational support is also relevant to the advocacy-ostracism relationship. Perceived organizational support refers to employees’ general belief that their organization values their contributions and cares about their well-being (Kurtessis et al., 2017). According to organizational support theory, employees who perceive stronger organizational support are more likely to experience psychological security, organizational trust, and confidence that valued work behaviors will be recognized rather than punished (Eisenberger and Stinglhamber, 2011). In nursing research, perceived organizational support has been associated with important work-related outcomes, including psychological capital, occupational stress, sleep problems, and well-being among Chinese nurses (Wang et al., 2022). These findings suggest that perceived organizational support may function as an organizational resource in ethically challenging clinical situations. In the context of patient advocacy, nurses who perceive higher organizational support may be more likely to believe that speaking up for patients is institutionally legitimate and protected. However, whether general organizational support can actually buffer the interpersonal risks associated with clinical advocacy remains unclear.

Overall, the existing literature reveals four important gaps. First, patient advocacy research has primarily emphasized its ethical legitimacy, professional value, and benefits for patient care, while giving limited attention to its possible interpersonal costs for nurses. Second, workplace ostracism research has documented many negative outcomes, but has paid less attention to ethically motivated antecedents in healthcare settings. Third, although moral courage is increasingly recognized as central to ethical nursing practice, its mediating role in the relationship between clinical advocacy behaviors and workplace ostracism has not been adequately tested. Fourth, perceived organizational support has been widely studied as a beneficial organizational resource, but its specific capacity to buffer advocacy-related ostracism remains uncertain. Addressing these gaps can deepen understanding of the hidden interpersonal costs of patient advocacy and provide a stronger empirical basis for developing organizational environments in which nurses can advocate for patients without incurring social exclusion.

### 1.5 Research model and hypotheses

**Figure 1:**
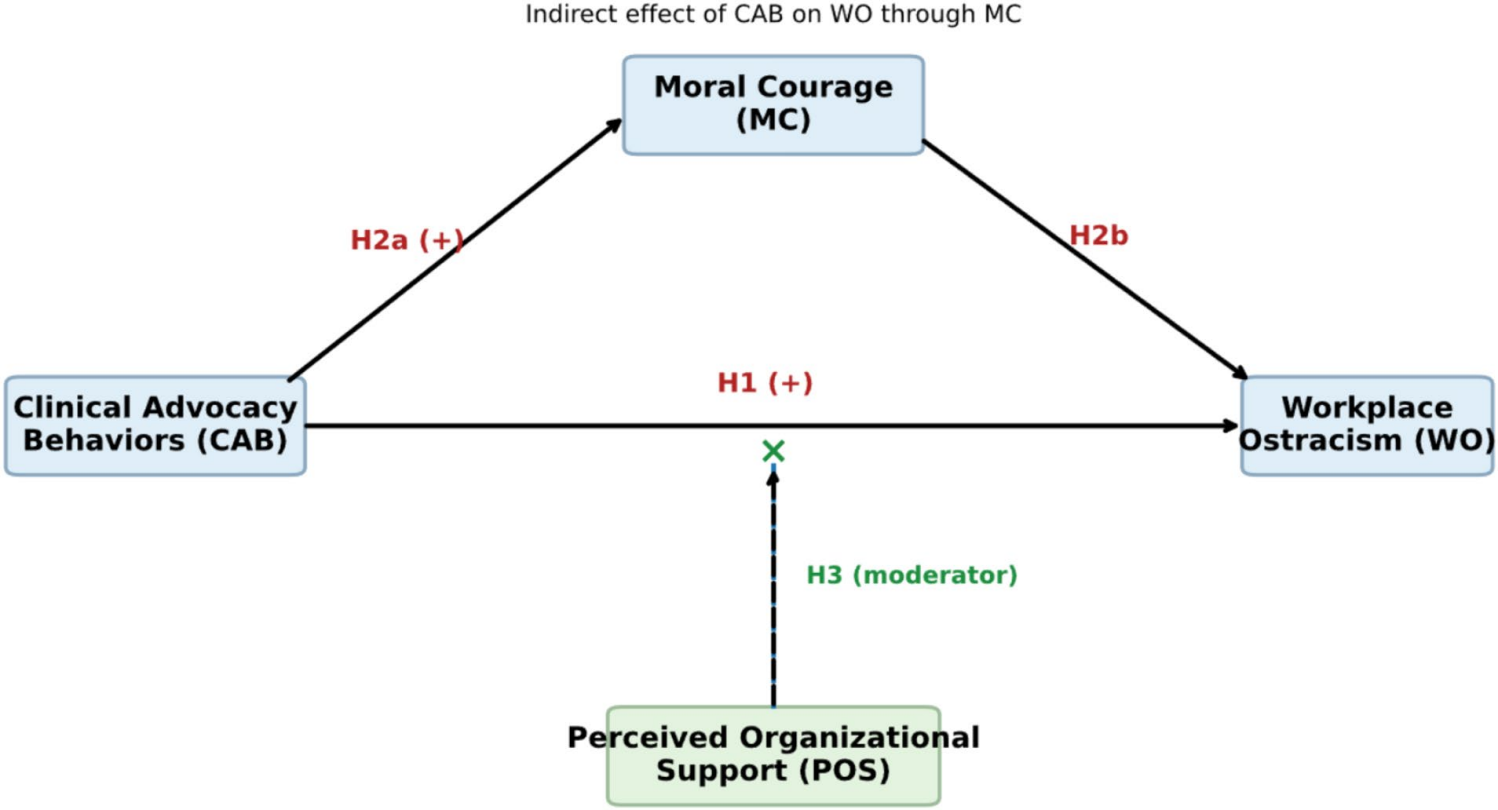
Proposed research model: Clinical advocacy behaviors (CAB) are hypothesized to be positively associated with workplace ostracism (WO), both directly and indirectly through moral courage (MC). Perceived organizational support (POS) is proposed to moderate the direct association between CAB and WO.

Based on the theoretical foundation and research mechanisms described above, this study proposes the research model shown in Figure 1. Specifically, CAB is expected to be positively associated with WO. This direct relationship is grounded in COR theory and Social Identity Theory, which suggest that advocacy may threaten nurses’ interpersonal resources and may be perceived as deviation from team norms. In addition, MC is proposed as a mediating mechanism explaining how CAB may translate into WO, whereas POS is proposed as a moderating mechanism indicating when the CAB-WO association may be weaker or stronger. Therefore, the study tests a moderated mediation-oriented conceptual model in which CAB is related to WO directly and indirectly through MC, and in which POS is expected to buffer the direct CAB-WO relationship.

First, CAB may increase nurses’ likelihood of experiencing WO. Although advocacy is ethically appropriate and professionally valued, it often involves questioning established routines, challenging authority gradients, or voicing concerns that may be perceived as disruptive within clinical teams. Such behaviors may threaten nurses’ interpersonal resources and position them as deviating from implicit group norms, thereby increasing the risk of exclusion in the workplace.

*Hypothesis 1 (H1)*: Clinical advocacy behaviors are positively associated with workplace ostracism among nurses.

Second, CAB is expected to be positively associated with MC. Advocacy frequently requires nurses to act in accordance with ethical principles despite uncertainty, interpersonal tension, or anticipated personal cost. Nurses who engage more actively in CAB may therefore exhibit higher levels of MC.

*Hypothesis 2a (H2a)*: Clinical advocacy behaviors are positively associated with moral courage.

Third, MC may function as an explanatory mechanism linking CAB to WO. Nurses with stronger MC are more likely to persist in ethically grounded action and to speak up in challenging situations. However, these same behaviors may also make them more visible as challengers of the status quo, thereby increasing their vulnerability to social exclusion.

*Hypothesis 2b (H2b)*: Moral courage mediates the relationship between clinical advocacy behaviors and workplace ostracism.

Finally, POS may buffer the interpersonal cost associated with CAB. When nurses perceive that their organization values their contributions and supports their well-being, advocacy is more likely to be interpreted as institutionally endorsed rather than personally risky. Under such conditions, the positive association between CAB and WO may be weakened.

*Hypothesis 3 (H3)*: Perceived organizational support moderates the relationship between clinical advocacy behaviors and workplace ostracism, such that the positive association is weaker when perceived organizational support is high.

## 2 Materials and methods

### 2.1 Study design and setting

This study employed a cross-sectional survey design to examine the relationships among clinical advocacy behaviors (CAB), moral courage (MC), workplace ostracism (WO), and perceived organizational support (POS). The study was conducted in three tertiary hospitals in Xi’an, Shaanxi Province, China, between October and November 2025.

### 2.2 Sample size estimation

The required sample size was estimated before data analysis using an *a priori* power analysis for multiple linear regression. Assuming a medium effect size (f^2^ = 0.15), a two-tailed significance level of *α* = 0.05, statistical power of 0.90, and up to 12 predictors in the most complex regression model, the minimum required sample size was approximately 157 participants. Because the study also planned mediation, moderation, and subgroup analyses, and because incomplete or invalid questionnaires were expected, the target sample size was increased to at least 300 participants. A total of 380 questionnaires were distributed, and 350 valid questionnaires were retained for analysis. The final sample size therefore exceeded the minimum requirement and was considered sufficient for the planned analyses.

### 2.3 Participants and sampling

A convenience sampling strategy was adopted to recruit registered nurses from the participating hospitals. Nurses were eligible if they: (a) were currently employed as registered nurses; (b) had at least 1 year of clinical work experience; (c) were engaged in direct patient care; and (d) were willing to provide informed consent. Nurses in administrative positions or those not involved in frontline clinical care were excluded.

A total of 380 questionnaires were distributed, of which 350 were returned and deemed valid for analysis, yielding a valid response rate of 92.1%.

### 2.4 Instruments

#### 2.4.1 Protective nursing advocacy scale (PNAS)

Clinical advocacy behaviors were measured using the Protective Nursing Advocacy Scale, originally developed by Hanks to assess nurses’ protective advocacy beliefs and actions (Hanks, 2005). The original scale contains 43 items across four dimensions: Advocate Actions, Work Context and Consequences, Environment and Educational Influences, and Support and Barriers to Advocacy. Items are rated on a 5-point Likert scale from 1 = strongly disagree to 5 = strongly agree. Total or mean scores can be calculated, with higher scores indicating stronger engagement in clinical advocacy behaviors. For the present study, the scale was translated into Chinese using forward translation and back-translation by bilingual nursing researchers. A panel of five senior nursing experts reviewed the translated version for semantic equivalence, cultural relevance, and content appropriateness. In the present sample, Cronbach’s *α* was 0.919.

#### 2.4.2 Moral courage scale for healthcare professionals (MCS-HP)

Moral courage was assessed using a 15-item adapted scale based on prior conceptual and empirical work on professional moral courage and moral courage in healthcare settings (Numminen et al., 2019). The adapted version included four dimensions: Moral Responsibility, Risk-Taking, Emotional Regulation, and Moral Persistence. Items were rated on a 5-point Likert scale, with higher scores indicating stronger moral courage. Because no universally established Chinese nursing version of this adapted instrument was available at the time of the study, the scale was translated and culturally adapted through forward-backward translation and expert review. The expert panel evaluated semantic clarity, clinical relevance, and cultural appropriateness for Chinese nurses. In the present sample, Cronbach’s *α* was 0.879.

#### 2.4.3 Workplace ostracism scale (WOS)

Workplace ostracism was measured using the 10-item Workplace Ostracism Scale developed by Ferris et al. (2008). The scale assesses the extent to which employees perceive being ignored, excluded, or left out by others at work. Items are rated on a 5-point scale from 1 = never to 5 = always. Higher mean scores indicate greater perceived workplace ostracism. The Chinese version used in this study was prepared through forward-backward translation and expert review to ensure linguistic and contextual equivalence for Chinese clinical nurses. In the present sample, Cronbach’s *α* was 0.786.

#### 2.4.4 Perceived organizational support scale (POS)

Perceived organizational support was measured using the 8-item short version of the Survey of Perceived Organizational Support, originally derived from (Eisenberger et al. (1997) organizational support theory and subsequent scale development work. Items assess employees’ general belief that the organization values their contributions and cares about their well-being. Items were rated on a 5-point Likert scale, with higher scores indicating stronger perceived organizational support. The scale was translated and adapted for Chinese nurses using forward-backward translation and expert review. In the present sample, Cronbach’s *α* was 0.718.

### 2.5 Data collection

Data collection was conducted after approval had been obtained from the Institutional Review Board of Northwest Women’s and Children’s Hospital (Approval No. 2025-NWH-IRB-042). Trained research assistants distributed paper-based questionnaires to eligible nurses during shift changes in the participating hospitals. Before participation, all nurses were informed of the study purpose, the voluntary nature of participation, and the measures taken to protect anonymity and confidentiality. Completion of the questionnaire was regarded as indicating informed consent. The average time required to complete the survey was approximately 15 to 20 min.

### 2.6 Statistical analysis

All statistical analyses were conducted using Python 3.12. The analytical procedure consisted of the following steps. First, descriptive statistics were calculated for demographic characteristics and study variables. Second, Pearson correlation analyses were performed to examine bivariate associations among CAB, MC, WO, and POS. Third, independent-samples t tests and one-way analysis of variance were used to examine differences in key variables across demographic and occupational subgroups. Fourth, hierarchical multiple regression analyses were conducted to test the hypothesized associations and to evaluate the incremental explanatory contribution of each set of variables.

Fifth, the mediating role of MC was examined using bootstrap analysis with 5,000 resamples to estimate bias-corrected confidence intervals for the indirect effect. This approach is widely recommended for mediation testing because it does not rely on the assumption of normality in the sampling distribution of indirect effects. The Sobel test was additionally reported as a supplementary reference. Sixth, subgroup analyses were performed by department and professional title to explore potential heterogeneity in the association between CAB and WO. Seventh, Harman’s single-factor test was used as a preliminary diagnostic for common method variance. However, given the known limitations of this approach, the results were interpreted cautiously and were not regarded as sufficient to definitively exclude common method bias. Finally, variance inflation factors (VIFs) were calculated to assess multicollinearity among predictors. All statistical tests were two-tailed, and a *p* value of less than 0.05 was considered statistically significant.

## 3 Results

### 3.1 Participant characteristics

**Figure 2:**
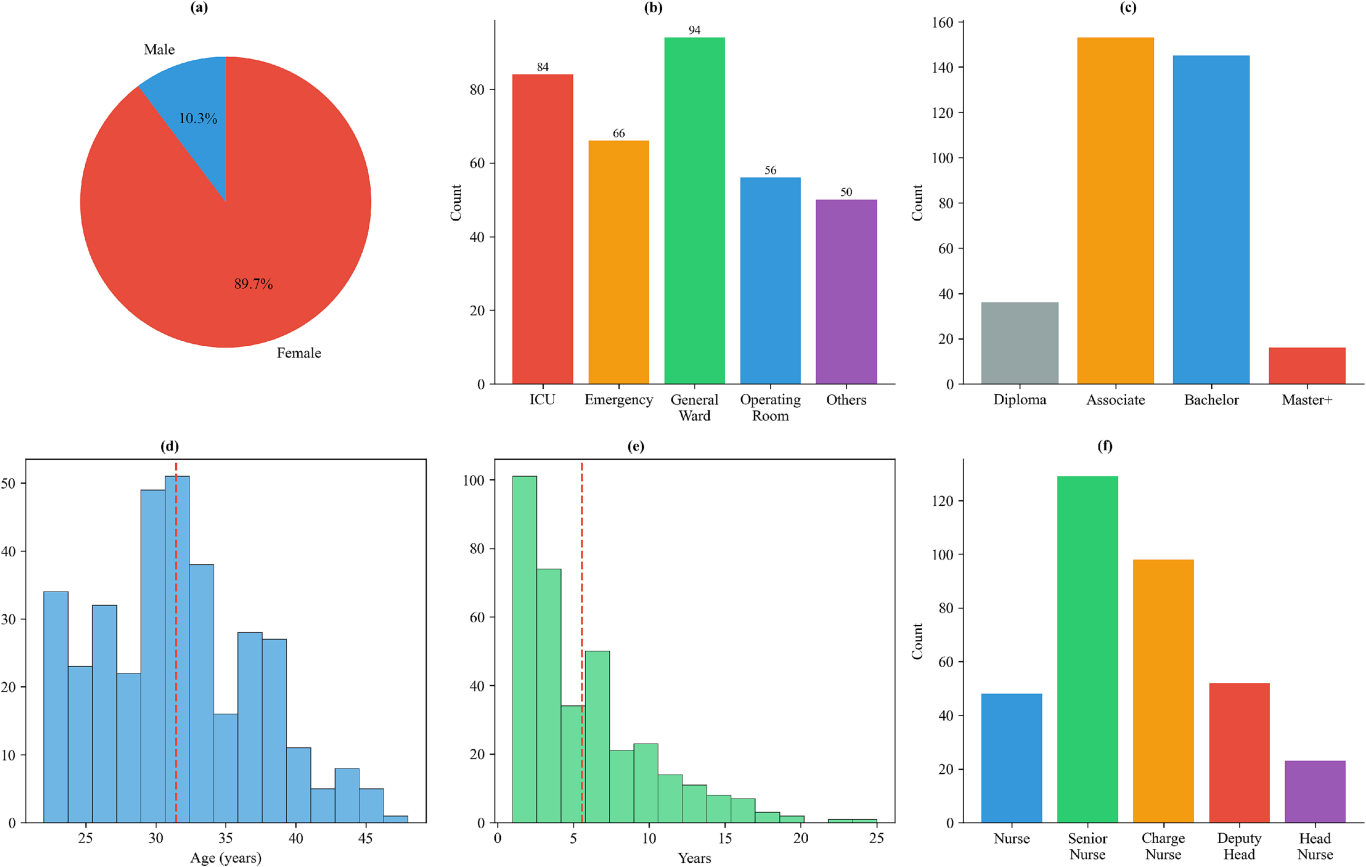
Distribution of participant characteristics (*N* = 350): **(a)** gender; **(b)** department; **(c)** education; **(d)** age distribution; **(e)** work experience; **(f)** professional title.

A total of 350 nurses were included in the final analysis. As shown in Figure 2, most participants were female (*n* = 314, 89.7%), whereas male nurses accounted for 10.3% of the sample (*n* = 36). The mean age of the participants was 31.5 years (SD = 5.7; range, 22–48 years), and the mean duration of work experience was 5.6 years (SD = 4.3).

Participants were drawn from a range of clinical departments. The largest proportion worked in general wards (*n* = 94, 26.9%), followed by intensive care units (ICU; *n* = 84, 24.0%), emergency departments (*n* = 66, 18.9%), operating rooms (*n* = 56, 16.0%), and other departments (*n* = 50, 14.3%).

In terms of educational attainment, most participants held an associate degree (*n* = 153, 43.7%) or a bachelor’s degree (*n* = 145, 41.4%), whereas relatively few had a diploma (*n* = 36, 10.3%) or a master’s degree or above (*n* = 16, 4.6%). Regarding professional title, senior nurses constituted the largest group (*n* = 129, 36.9%), followed by charge nurses (*n* = 98, 28.0%), deputy head nurses (*n* = 52, 14.9%), nurses (*n* = 48, 13.7%), and head nurses (*n* = 23, 6.6%).

Overall, the sample was composed primarily of early- to mid-career female nurses working in general wards and critical care-related settings, with most participants holding associate or bachelor’s degrees.

### 3.2 Descriptive statistics

Table 1 presents the descriptive statistics, distributional indices, and internal consistency estimates for the four main study variables. CAB had a mean score of 3.17 (SD = 0.45), indicating a moderate-to-high level of advocacy engagement. MC had a mean of 3.07 (SD = 0.50), suggesting a moderate level of moral courage. WO showed a relatively low mean score (M = 1.84, SD = 0.42), indicating that workplace ostracism was not highly prevalent overall in this sample. POS had a mean of 2.85 (SD = 0.57), reflecting a moderate level of perceived organizational support.

**Table 1:** Descriptive statistics of main study variables (*N* = 350).

Variable	Items	M	SD	Min	Max	Skewness	Kurtosis	Cronbach’s α
CAB (PNAS)	43	3.166	0.454	2.05	4.58	−0.032	0.125	0.919
MC (MCS-HP)	15	3.073	0.496	1.67	4.80	0.077	0.238	0.879
WO (WOS)	10	1.837	0.415	1.00	3.10	0.264	−0.289	0.786
POS	8	2.847	0.569	1.25	4.50	0.053	−0.026	0.718

All variables showed adequate dispersion, with observed values spanning a reasonable range. Skewness and kurtosis values were close to zero for all measures, suggesting approximate normality. In addition, the internal consistency of the four scales ranged from acceptable to excellent, with Cronbach’s alpha coefficients between 0.718 and 0.919.

**Figure 3:**
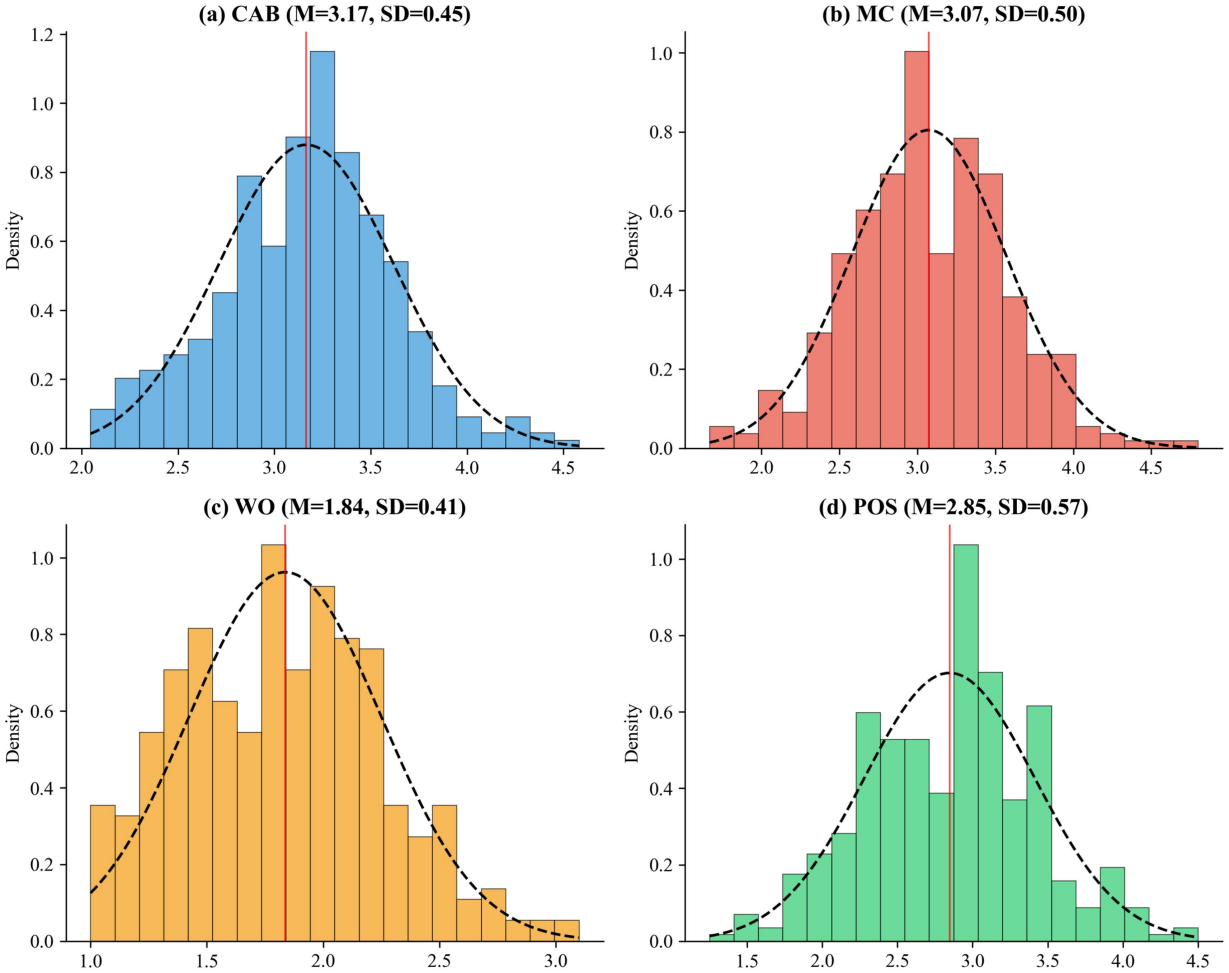
Distribution of main study variables with normal curve overlay. **(a)** CAB; **(b)** MC; **(c)** WO; **(d)** POS.

The reliability analysis indicated that all four instruments demonstrated acceptable to excellent internal consistency. As shown in Figure 3, Cronbach’s alpha coefficients were 0.919 for clinical advocacy behaviors (CAB), 0.879 for moral courage (MC), 0.786 for workplace ostracism (WO), and 0.718 for perceived organizational support (POS). These values exceeded the commonly accepted threshold of 0.70, supporting the internal consistency of the study measures. In particular, the CAB and MC scales showed strong reliability, whereas the WO and POS scales demonstrated acceptable reliability for research purposes.

To preliminarily assess the potential influence of common method variance, Harman’s single-factor test was conducted. The first unrotated factor accounted for 18.45% of the total variance, which was well below the conventional 40% threshold. This result suggests that a single latent factor did not account for the majority of covariance among the measured variables, thereby providing no strong indication that common method variance seriously distorted the data structure. Nevertheless, because Harman’s single-factor test is widely recognized as a relatively insensitive diagnostic tool, its result should be interpreted cautiously and cannot be taken as definitive evidence that common method bias was absent. This issue is therefore acknowledged as a methodological limitation of the present study.

Multicollinearity was further examined using variance inflation factors (VIFs). The VIF values for CAB, MC, and POS were 1.861, 1.859, and 1.002, respectively, all of which were substantially below the conventional cutoff value of 10. These findings indicate that multicollinearity was not a concern in the regression analyses and that the estimated coefficients were unlikely to be distorted by excessive overlap among predictors.

Overall, the reliability and diagnostic results support the adequacy of the measurement properties for the subsequent correlational, regression, and mediation analyses.

### 3.3 Correlation analysis

Table 2 presents the Pearson correlation coefficients among the core study variables and selected demographic variables. As hypothesized, clinical advocacy behaviors (CAB) were significantly and positively correlated with moral courage (MC) (*r* = 0.679, *p* < 0.001) and workplace ostracism (WO) (*r* = 0.360, *p* < 0.001). MC was also significantly positively correlated with WO (*r* = 0.335, *p* < 0.001). These findings provide preliminary support for the proposed model, suggesting that nurses who reported higher levels of CAB also tended to report stronger MC and greater WO, and that MC was likewise associated with higher levels of WO.

**Table 2:** Pearson correlation matrix (*N* = 350).

Variable	1	2	3	4	5	6
1. CAB	1					
2. MC	0.679***	1				
3. WO	0.360***	0.335***	1			
4. POS	0.031	−0.004	−0.057	1		
5. Age	0.027	0.079	0.034	0.097	1	
6. Experience	0.057	0.087	−0.011	−0.059	0.023	1

***p < 0.001, **p < 0.01, *p < 0.05.

By contrast, perceived organizational support (POS) was not significantly correlated with CAB (*r* = 0.031, *p* > 0.05), MC (*r* = −0.004, *p* > 0.05), or WO (*r* = −0.057, *p* > 0.05). Although the association between POS and WO was negative in direction, its magnitude was very small and did not reach statistical significance. This pattern suggests that POS did not show a meaningful zero-order association with the focal variables in the present sample, which is consistent with the later finding that its moderating effect was not statistically significant.

**Figure 4:**
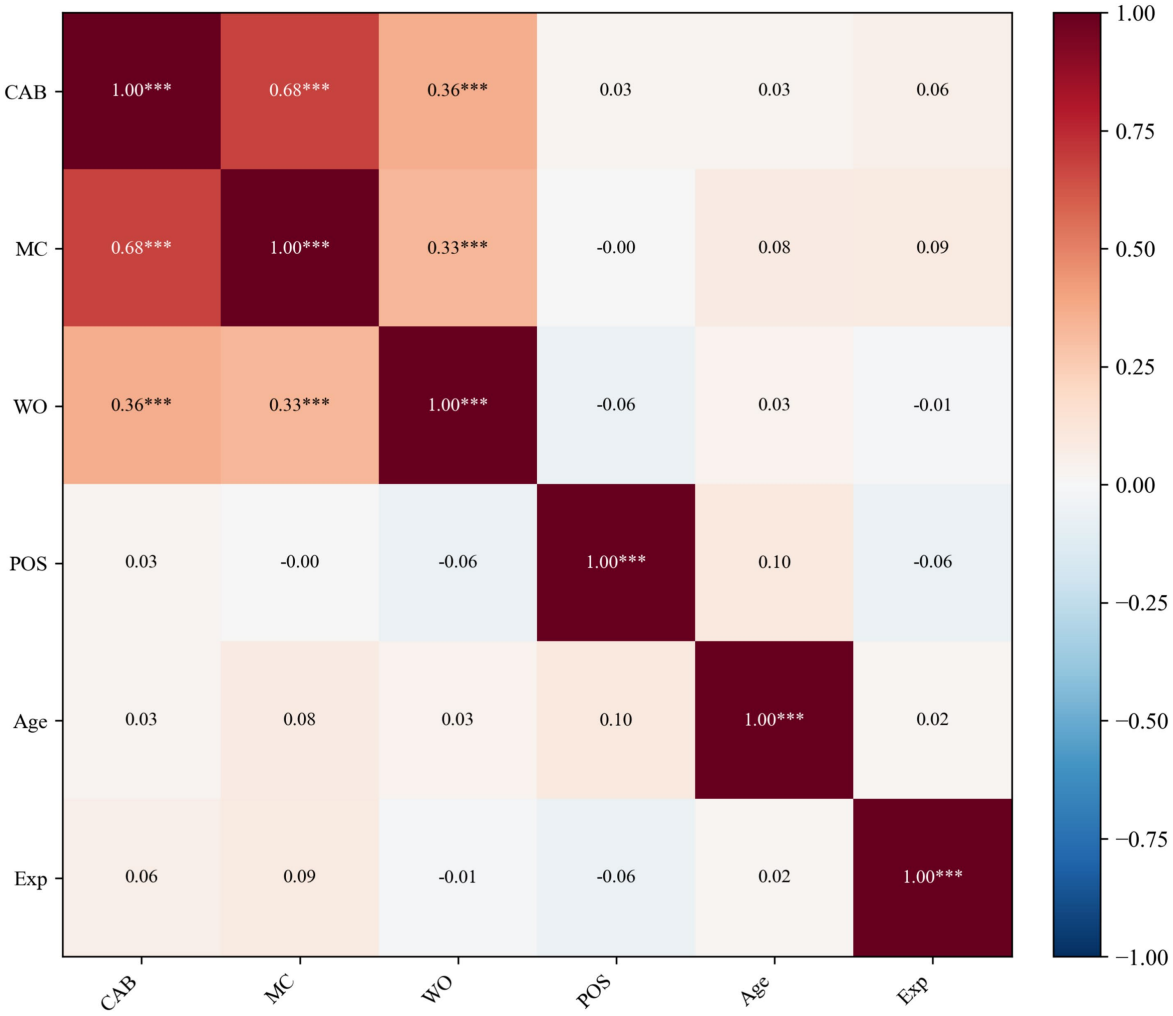
Correlation heatmap of study variables.

Age and work experience were only weakly correlated with the main study variables, with all coefficients close to zero. These results suggest that the associations among CAB, MC, and WO were not substantially driven by basic demographic or career-stage differences (see Figure 4).

**Figure 5:**
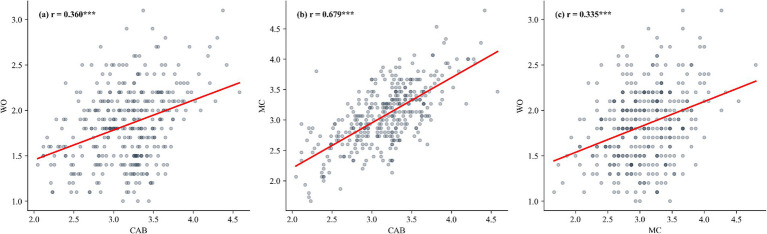
Bivariate scatter plots with regression lines for key variable pairs. **(a)** CAB and WO; **(b)** CAB and MC; **(c)** MC and WO.

Figure 5 provides a heatmap visualization of the correlation matrix. The figure clearly shows that the strongest association in the model was between CAB and MC, followed by the moderate positive associations between CAB and WO and between MC and WO. In contrast, the cells involving POS, age, and work experience were much lighter in color, indicating near-zero correlations with the focal constructs. This visual pattern reinforces the interpretation that the core empirical structure of the data is centered on the interrelationships among CAB, MC, and WO, rather than on demographic background or perceived organizational support.

**Figure 6:**
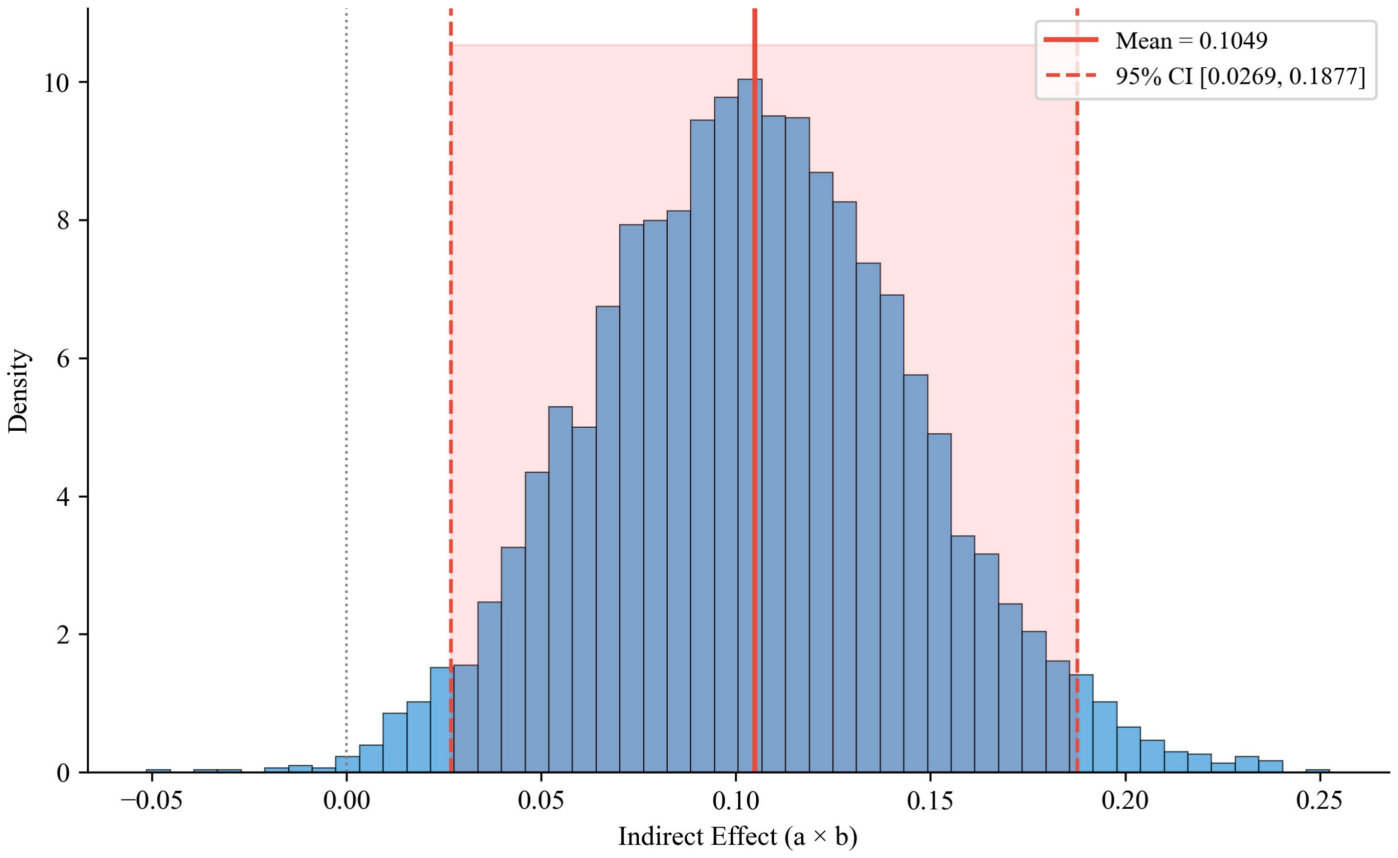
Bootstrap distribution of the indirect effect (5,000 resamples). The 95% CI does not contain zero.

Figure 6 further illustrates the bivariate relationships among the three key variable pairs using scatter plots with fitted regression lines. Panel (a) shows a positive linear association between CAB and WO, indicating that higher advocacy engagement tended to be accompanied by higher levels of perceived workplace ostracism. Although the slope is not steep, the upward trend is clear, and the data points are distributed around the fitted line without obvious evidence of strong nonlinearity. Panel (b) shows the strongest linear pattern, between CAB and MC, with points clustering closely around the regression line, consistent with the relatively large correlation coefficient (r = 0.679). This suggests that nurses who engaged more strongly in advocacy also tended to report markedly higher levels of moral courage. Panel (c) shows a more modest but still clear positive relationship between MC and WO, indicating that greater moral courage was associated with greater workplace ostracism.

Importantly, the scatter plots do not reveal severe outliers, obvious curvilinear patterns, or substantial heteroscedasticity. Instead, the relationships appear approximately linear and reasonably well behaved, supporting the appropriateness of subsequent parametric analyses, including hierarchical regression and mediation testing. Taken together, the tabular and graphical results consistently support the hypothesized direct associations among CAB, MC, and WO and provide an empirical basis for the regression and mediation analyses reported in the following sections.

### 3.4 Hierarchical regression analysis

Table 3 presents the results of the hierarchical regression analysis predicting workplace ostracism (WO). In Step 1, demographic variables were entered as controls and explained 2.7% of the variance in WO (R^2^ = 0.027), indicating that basic background characteristics contributed only modestly to differences in perceived workplace ostracism.

**Table 3:** Hierarchical regression analysis predicting workplace ostracism.

Predictor	Step 1 β	Step 2 β	Step 3 β	Step 4 β
Demographics	Controlled	Controlled	Controlled	Controlled
CAB		0.373***	0.251***	0.257***
MC			0.181**	0.177**
POS				−0.064
CAB × POS				−0.084
*R* ^2^	0.027	0.163	0.180	0.188

***p < 0.001, **p < 0.01. Standardized coefficients reported.

In Step 2, clinical advocacy behaviors (CAB) were added to the model. CAB was a significant positive predictor of WO (*β* = 0.373, *t* = 7.430, *p* < 0.001), and its inclusion increased the explained variance by 13.6% (ΔR^2^ = 0.136, *p* < 0.001). This substantial increment indicates that CAB accounted for a meaningful proportion of variance in WO above and beyond demographic factors. The positive coefficient suggests that nurses who reported higher levels of advocacy engagement were also more likely to report workplace ostracism. These findings provide support for Hypothesis 1.

In Step 3, moral courage (MC) was entered to test its additional explanatory contribution. MC was positively associated with WO (*β* = 0.181, *t* = 2.665, *p* = 0.008), and the model explained an additional 1.7% of the variance (ΔR^2^ = 0.017, *p* < 0.01). Notably, the standardized coefficient for CAB decreased from 0.373 to 0.251 after MC was added, representing a 32.7% reduction in effect size. This attenuation suggests that part of the association between CAB and WO may be transmitted through MC, thereby providing preliminary evidence for partial mediation.

In Step 4, perceived organizational support (POS) and the interaction term between CAB and POS were added to examine moderation. Neither POS nor the interaction term reached statistical significance, indicating that POS did not significantly alter the strength of the relationship between CAB and WO. Although the full model explained 18.8% of the variance in WO (R^2^ = 0.188), the additional variance explained at Step 4 was small and non-significant (ΔR^2^ = 0.007). These results suggest that the positive association between CAB and WO was relatively stable across levels of perceived organizational support, and thus Hypothesis 3 was not supported.

Overall, the hierarchical regression results indicate that CAB was a robust positive predictor of WO, MC contributed additional explanatory value and reduced the magnitude of the CAB coefficient, and POS did not exert a significant moderating effect. This pattern is consistent with a model in which MC partially explains, but POS does not significantly buffer, the interpersonal cost associated with clinical advocacy behaviors.

### 3.5 Mediation analysis

The mediating role of moral courage (MC) in the association between clinical advocacy behaviors (CAB) and workplace ostracism (WO) was examined using bootstrap analysis with 5,000 resamples. As shown in Table 4, CAB was significantly and positively associated with MC (path a: *β* = 0.743, *t* = 17.269, *p* < 0.001), providing strong support for Hypothesis 2a. This result indicates that nurses who reported greater engagement in clinical advocacy behaviors also tended to report substantially higher levels of moral courage.

**Table 4:** Mediation analysis results (Bootstrap, 5,000 resamples).

Path	Effect	SE	*t*	*p*	95% CI
a (CAB → MC)	0.743	0.043	17.269	<0.001	[0.658, 0.827]
b (MC → WO)	0.140	0.057	2.481	0.014	[0.029, 0.252]
c (total)	0.329	0.046	7.194	<0.001	[0.239, 0.418]
c′ (direct)	0.225	0.062	3.633	<0.001	[0.103, 0.346]
a × b (indirect)	0.105	0.041	—	—	[0.027, 0.188]
Sobel Z	2.456	—	—	0.014	—
Mediation %	32.0%	—	—	—	—

MC, in turn, was significantly positively associated with WO after controlling for CAB (path b: *β* = 0.140, *t* = 2.481, *p* = 0.014). This finding suggests that higher levels of moral courage were associated with greater perceived workplace ostracism, even after accounting for the direct contribution of CAB. The total effect of CAB on WO was significant (path c: *β* = 0.329, *t* = 7.194, *p* < 0.001), indicating that CAB was positively associated with WO before the mediator was included in the model. After MC was entered, the direct effect of CAB on WO remained significant but was reduced in magnitude (path c′: *β* = 0.225, *t* = 3.633, *p* < 0.001).

The indirect effect of CAB on WO through MC was 0.105. Bootstrap analysis showed that the 95% bias-corrected confidence interval for this indirect effect was 0.027 to 0.188, which did not include zero, indicating a statistically significant mediation effect. This indirect pathway accounted for 32.0% of the total effect, suggesting that approximately one-third of the association between CAB and WO operated through MC. At the same time, because the direct effect (c′) remained statistically significant after inclusion of the mediator, the results indicate partial rather than full mediation.

As a supplementary check, the Sobel test also yielded a significant result (*Z* = 2.456, *p* = 0.014), which was consistent with the bootstrap findings. Given that bootstrap confidence intervals are generally regarded as the more robust criterion for testing indirect effects, greater interpretive weight was placed on the bootstrap results. Overall, these findings support Hypothesis 2b and suggest that MC serves as an important, though not exclusive, mechanism linking CAB to WO.

Figure 6 presents the bootstrap distribution of the indirect effect. The empirical distribution was centered above zero, and the corresponding 95% confidence interval did not cross zero, visually reinforcing the conclusion that the indirect effect was statistically significant.

### 3.6 Subgroup analysis

To further examine whether the association between clinical advocacy behaviors (CAB) and workplace ostracism (WO) varied across occupational contexts, subgroup analyses were conducted by department and professional title. The results revealed notable heterogeneity in the strength of the CAB-WO association across both subgroup classifications.

#### 3.6.1 By department

As shown in Table 5, the magnitude of the association between CAB and WO differed across departments. The strongest positive correlation was observed among nurses working in the emergency department (*r* = 0.428, *p* < 0.001), followed closely by those in the intensive care unit (ICU; *r* = 0.417, *p* < 0.001) and in other departments (*r* = 0.408, *p* = 0.003). A moderate positive association was also found among nurses in general wards (*r* = 0.318, *p* = 0.002). In contrast, the association was weaker and not statistically significant among operating room nurses (*r* = 0.182, *p* = 0.180).

**Table 5:** Subgroup analysis of the association between clinical advocacy behaviors and workplace ostracism by department.

Department	*n*	*r*	*p-value*
ICU	84	0.417***	<0.001
Emergency	66	0.428***	<0.001
General ward	94	0.318**	0.002
Operating room	56	0.182	0.180
Others	50	0.408**	0.003

***p < 0.001, **p < 0.01. Standardized coefficients reported.

Overall, these findings suggest that the positive CAB-WO relationship was more pronounced in high-intensity and fast-paced clinical environments, particularly emergency and critical care settings. By contrast, the weaker association observed in the operating room may indicate that the interpersonal consequences of advocacy differ across departmental cultures, workflow structures, or team interaction patterns.

#### 3.6.2 Subgroup analysis by professional title

As shown in Table 6, substantial variation was also observed across professional titles. The strongest association between CAB and WO was found among entry-level nurses (*r* = 0.602, *p* < 0.001), indicating a relatively strong positive relationship in this subgroup. Significant positive associations were also observed among charge nurses (*r* = 0.444, *p* < 0.001), head nurses (*r* = 0.428, *p* = 0.042), and senior nurses (*r* = 0.239, *p* = 0.006). By contrast, the association was weaker and not statistically significant among deputy head nurses (*r* = 0.182, *p* = 0.197).

**Table 6:** Subgroup analysis by professional title.

Title	*n*	*r*	*p-value*
Nurse	48	0.602***	<0.001
Senior nurse	129	0.239**	0.006
Charge nurse	98	0.444***	<0.001
Deputy head	52	0.182	0.197
Head nurse	23	0.428*	0.042

***p < 0.001, **p < 0.01. Standardized coefficients reported.

This pattern suggests that the interpersonal cost associated with CAB may be particularly salient among nurses at earlier career stages, who may have less organizational authority, fewer relational resources, or weaker protection against exclusionary responses. Although significant associations were also observed among some senior positions, the especially strong correlation among entry-level nurses highlights their potential vulnerability within workplace hierarchies.

Taken together, the subgroup analyses indicate that the positive association between CAB and WO was not uniform across occupational contexts. Rather, it appeared stronger in certain departments and professional strata, especially among emergency nurses, ICU nurses, and entry-level nurses. These findings suggest that the interpersonal consequences of advocacy may be shaped by both local work environments and hierarchical position within the nursing workforce.

### 3.7 Moderation analysis

The moderating role of perceived organizational support (POS) was examined by testing interaction terms in the regression model. As shown in the full model, the interaction between clinical advocacy behaviors (CAB) and POS was not statistically significant (*β* = 0.053, *p* = 0.437), indicating that POS did not significantly moderate the association between CAB and workplace ostracism (WO). Accordingly, Hypothesis 3 was not supported. An additional exploratory interaction involving MC was also tested but was not significant.

Taken together, these findings indicate that the hypothesized moderation component was not confirmed. Although the mediation analysis supported the indirect role of MC, there was no evidence that the relationship between CAB and WO varied as a function of POS. Because the relevant interaction terms were non-significant, a formal test of conditional indirect effects, such as an index of moderated mediation, was not pursued. This analytic decision was made to avoid overinterpreting a moderated mediation structure that lacked empirical support at the interaction level.

**Figure 7:**
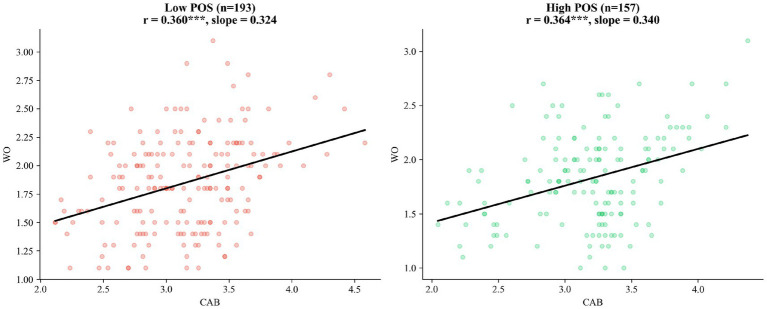
CAB–WO relationship by POS level.

To further illustrate the absence of moderation, an exploratory median-split analysis was conducted by dividing participants into low-POS and high-POS groups. As shown in Figure 7, the correlation between CAB and WO was highly similar in the low-POS group (*r* = 0.360) and the high-POS group (*r* = 0.364). The fitted regression lines in the two panels also showed nearly identical slopes, providing visual confirmation that the CAB-WO relationship remained largely stable across levels of POS. This graphical pattern is consistent with the non-significant interaction results and further supports the conclusion that POS did not exert a meaningful buffering effect in the present sample.

### 3.8 Model diagnostics

Model diagnostics were conducted to assess whether the assumptions underlying the regression analyses were reasonably satisfied. Examination of the residuals-versus-fitted plots did not reveal any obvious systematic pattern, suggesting that the assumptions of linearity and homoscedasticity were broadly met. In addition, the normal Q-Q plots indicated that the residuals approximated a normal distribution, with no substantial deviations at the tails. Histogram-based inspection of the residuals similarly suggested approximate normality.

**Figure 8:**
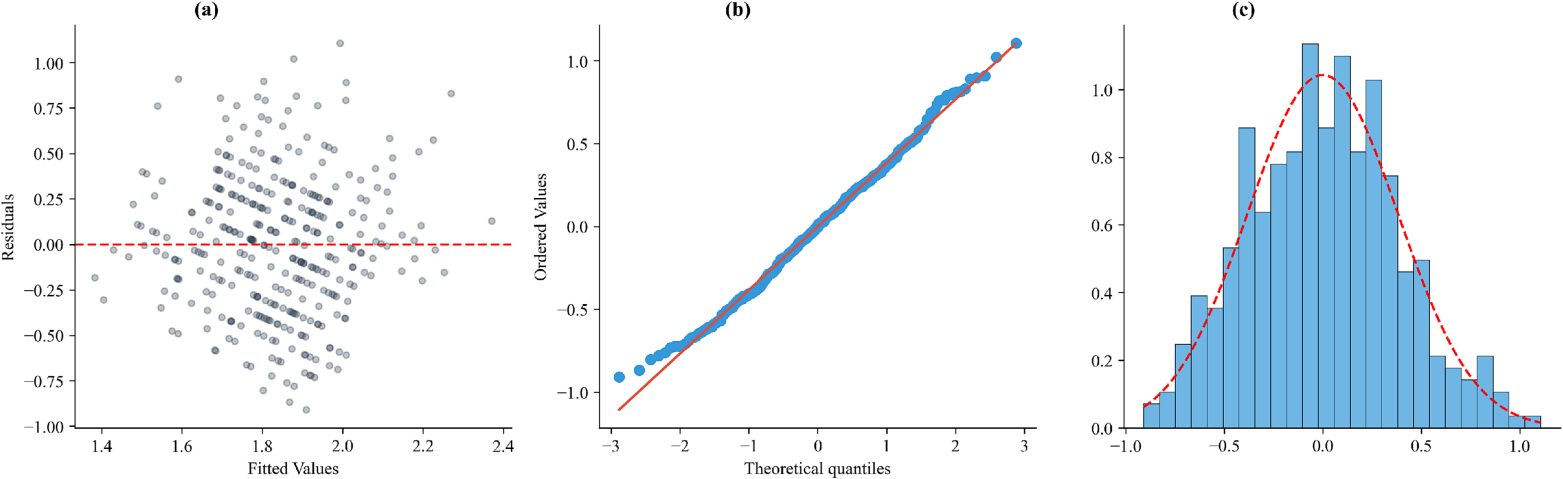
Model diagnostic plots for the full regression model: **(a)** residuals versus fitted values; **(b)** normal Q-Q plot; **(c)** residual distribution.

Taken together, these diagnostic results support the adequacy of the regression model and suggest that the main parameter estimates were unlikely to be materially distorted by major violations of model assumptions. Figure 8 presents the residual diagnostic plots for the full regression model.

**Figure 9:**
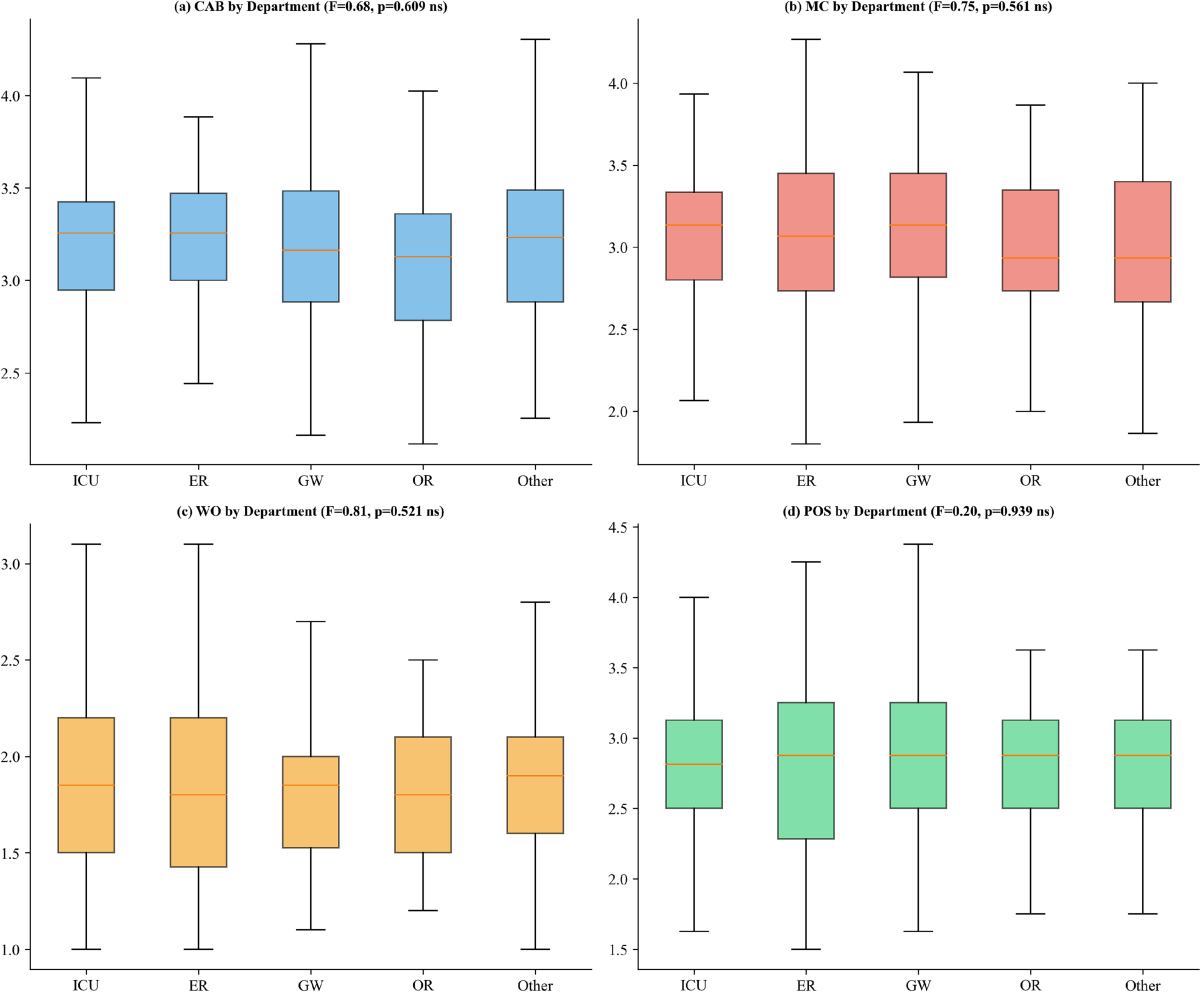
Mean differences in the main study variables across departments. Mean differences in the main study variables across departments: **(a)** CAB; **(b)** MC; **(c)** WO; **(d)** POS.

To further examine departmental heterogeneity, Figure 9 displays the mean levels of the main study variables across departments. The visual pattern suggests that nurses from different departments varied not only in the strength of the CAB-WO association but also in the overall levels of CAB, MC, WO, and POS. Notably, emergency and ICU settings appeared to show relatively greater variation in WO and related constructs, whereas the operating room showed a comparatively weaker pattern. These descriptive results complement the subgroup correlation analysis and reinforce the interpretation that departmental context may influence both the prevalence and interpersonal consequences of advocacy-related behaviors.

## 4 Discussion

This study examined the association between clinical advocacy behaviors (CAB) and workplace ostracism (WO) among Chinese nurses, and further tested moral courage (MC) as a mediating mechanism and perceived organizational support (POS) as a moderating condition. Four main findings emerged. First, CAB was positively associated with WO, indicating that patient advocacy may carry hidden interpersonal costs for nurses. Second, MC partially mediated this association, suggesting that advocacy may translate into ostracism partly through nurses’ morally courageous action. Third, the CAB-WO association varied across departments and professional titles, with stronger associations among emergency nurses, ICU nurses, and entry-level nurses. Fourth, POS did not significantly moderate the association between CAB and WO. These findings extend the nursing advocacy literature by shifting attention from the patient-centered benefits of advocacy to its possible relational consequences for nurses.

### 4.1 The hidden interpersonal cost of patient advocacy

The present study showed that CAB was positively associated with WO, supporting H1. This finding suggests that patient advocacy may involve not only ethical and clinical value, but also measurable interpersonal risk for nurses. Previous advocacy research has mainly emphasized the protective role of nurses in defending patients’ rights, promoting informed decision-making, preventing harm, and improving care quality. The current findings add a relational dimension to this literature by showing that advocacy may also expose nurses to exclusion, distancing, or other subtle negative social responses.

This result is consistent with research on speaking up for patient safety, which shows that healthcare professionals often hesitate to raise concerns because speaking up is shaped by perceived risk, hierarchy, team climate, and expected reactions from others (Okuyama et al., 2014). CAB may operate in a similar way. When nurses advocate for patients by questioning clinical decisions, interrupting routines, or calling attention to problems, these behaviors may be interpreted by coworkers or supervisors as disruptive rather than constructive. From the perspective of Conservation of Resources theory, advocacy may threaten nurses’ relational resources, including collegial support, professional reputation, and a sense of interpersonal security. From the perspective of Social Identity Theory, advocacy may also be perceived as a deviation from implicit team norms, especially when it challenges authority gradients or established patterns of coordination.

The practical significance of this finding should also be emphasized. The positive CAB-WO association indicates a paradox for nursing ethics and management: behaviors that protect patients may simultaneously weaken the social relationships that help nurses function effectively within teams. Prior survey evidence has shown that healthcare professionals may withhold patient safety concerns because they anticipate negative interpersonal consequences, doubt that speaking up will be effective, or fear damaging relationships with colleagues and supervisors (Schwappach and Gehring, 2015). Therefore, advocacy should not be understood only as an individual ethical obligation. It should also be understood as a socially situated behavior whose consequences depend on whether the surrounding team environment tolerates disagreement and protects voice.

This paradox may be particularly relevant in the Chinese healthcare context, but this interpretation should be grounded in institutional and empirical evidence rather than broad cultural generalization. China has a large nursing workforce, with 5.63 million registered nurses by the end of 2023 and approximately four registered nurses per 1,000 population (Xinhua News Agency, 2024). Many nurses in tertiary hospitals work under high patient volume, intensive interprofessional coordination, and strong role-based hierarchies. Recent evidence also indicates that Chinese nurses face substantial workplace interpersonal strain, including workplace psychological violence and other relational stressors (Luo et al., 2024). Against this background, patient advocacy may become socially sensitive when it requires nurses to express disagreement upward, challenge routine practices, or cross professional boundaries. The present findings therefore suggest that the interpersonal cost of advocacy deserves closer empirical attention in Chinese nursing settings.

### 4.2 The mediating role of moral courage

MC partially mediated the association between CAB and WO, accounting for 32.0% of the total effect. This finding identifies one pathway through which advocacy may translate into interpersonal cost. Nurses who reported stronger advocacy behaviors also reported higher MC, which is conceptually coherent because advocacy often requires ethical clarity, persistence, and willingness to act despite uncertainty or possible disadvantage. A recent study of Chinese nurses similarly showed that moral courage is a clinically relevant professional attribute associated with work experience, income, organizational ethics training, and other contextual factors (Huang et al., 2023). The present study extends this line of research by showing that MC is not only related to ethical nursing practice, but also helps explain why advocacy may be associated with ostracism.

At the same time, the positive association between MC and WO reveals a complex pattern. MC is usually framed as a desirable ethical capacity, but morally courageous action may also make nurses more visible as individuals who challenge unsafe practices, question decisions, or resist inappropriate routines. A recent systematic review concluded that nurses’ moral courage is shaped by individual, moral, and organizational factors, indicating that moral courage does not operate in isolation from the work environment (Abdollahi et al., 2024). In the present study, this helps explain why MC may carry relational consequences. Nurses with higher MC may be more willing to act on ethical concerns, but such action may also be interpreted by others as confrontational or norm-challenging.

The mediation effect was partial rather than complete, indicating that MC explains an important but not exhaustive part of the CAB-WO relationship. Other mechanisms may also be involved, such as direct interpersonal conflict, perceived threats to colleagues’ competence, workflow interruption, role-boundary tension, or differences in leader responsiveness. Qualitative research on speaking-up behavior in healthcare has shown that clinicians’ decisions to voice concerns are influenced by multiple individual, relational, and contextual factors, including hierarchy, audience receptiveness, urgency, perceived safety, and expected consequences (Umoren et al., 2022). Future research should therefore examine additional mediating pathways and use longitudinal designs to clarify the temporal ordering among CAB, MC, and WO.

### 4.3 Subgroup heterogeneity

One notable finding was the heterogeneity in the CAB-WO association across departments and professional titles. The association was stronger among emergency and ICU nurses, but weaker and non-significant among operating room nurses. This pattern may reflect differences in clinical urgency, workflow structure, communication norms, and team routines across departments. Emergency and ICU settings are characterized by time pressure, uncertainty, rapid decision-making, and high interdependence. Under such conditions, advocacy may be more likely to be perceived as interruptive if it delays action or challenges an ongoing decision. In contrast, operating room work is often more protocolized, and some forms of advocacy may be normalized through standardized safety procedures. Evidence from the surgical safety checklist literature suggests that structured communication tools can improve consistency of care and strengthen team communication, which may help legitimize speaking up in perioperative settings (Haynes et al., 2009).

Heterogeneity by professional title was also informative. Entry-level nurses showed the strongest CAB-WO association, whereas the association was weaker and non-significant among deputy head nurses. This suggests that organizational position and symbolic authority may shape how advocacy is interpreted. For junior nurses, advocacy may be seen as overstepping role boundaries or challenging more senior staff without sufficient legitimacy. For more senior nurses, similar behaviors may be interpreted as expected professional responsibility or legitimate supervisory input. This interpretation is consistent with reviews of employee voice and silence in healthcare, which emphasize that speaking up is shaped by hierarchy, professional status, leadership responsiveness, psychological safety, and organizational culture (Lainidi et al., 2023). The present findings therefore suggest that the interpersonal cost of advocacy is not evenly distributed across the nursing workforce. It may fall disproportionately on nurses with less authority and fewer relational resources.

### 4.4 The non-significant moderation of perceived organizational support

Contrary to H3, POS did not significantly moderate the association between CAB and WO. This result indicates that the positive association between advocacy and ostracism remained relatively stable across levels of general organizational support. Although MC helped explain how CAB was associated with WO, POS did not function as a significant buffer in this sample.

Several explanations are possible. First, advocacy-related ostracism may operate primarily through informal interpersonal channels, such as peer distancing, selective exclusion, withholding information, or subtle withdrawal of social acceptance. These processes may not be easily offset by nurses’ general perception that the organization values them. Second, POS may be too broad to capture the specific forms of support that matter when nurses face advocacy-related relational risk. More proximal resources, such as psychologically safe team climates, inclusive leadership, supervisor protection, peer allyship, and protected escalation procedures, may be more relevant. Psychological safety theory suggests that what matters for interpersonal risk-taking is not only general organizational support, but also a shared team-level belief that members can raise concerns, ask questions, and challenge routines without fear of punishment or humiliation (Edmondson, 1999). This may explain why general POS did not significantly weaken the CAB-WO association in the present study.

Accordingly, future research should move beyond global POS and examine more specific boundary conditions. These may include unit-level safety climate, leader inclusiveness, voice-specific supervisory support, moral climate, peer support, and formal protection mechanisms for advocacy. Multilevel research designs would be particularly useful because advocacy and ostracism may unfold at the intersection of individual courage, team norms, and organizational structures.

### 4.5 Practical implications

Several practical implications follow from the present findings. First, healthcare organizations should recognize that patient advocacy may carry interpersonal risk and that this risk may remain invisible if attention is limited to overt conflict or formal complaints. Hospitals should therefore establish protected advocacy pathways that allow nurses to raise patient-related concerns without fear of informal retaliation or social exclusion. These pathways may include confidential reporting channels, clearly defined escalation procedures, written feedback from managers, and explicit non-retaliation rules.

Second, high-acuity departments such as emergency units and ICUs should implement structured speak-up routines. Examples include safety huddles, brief pre-shift risk discussions, post-event debriefings, standardized communication scripts, and explicit leader invitations to raise concerns. TeamSTEPPS tools such as the Two-Challenge Rule are designed to support team members in speaking up when their concerns are not initially acknowledged, and such tools may help transform advocacy from an individual act of confrontation into a shared team practice (Agency for Healthcare Research and Quality, 2024).

Third, junior nurses require targeted protection. Because entry-level nurses showed the strongest CAB-WO association, mentorship and senior sponsorship should be incorporated into advocacy-related management strategies. Junior nurses should have access to experienced nurses who can help them formulate concerns, select appropriate communication channels, and provide relational support after difficult advocacy events. This is particularly important in departments where hierarchy and time pressure may make direct advocacy more socially risky.

Fourth, nurse managers should be trained to identify and interrupt subtle ostracism. Relevant behaviors include excluding a nurse from informal communication, withholding work-related information, avoiding collaboration after a nurse raises a concern, or framing advocacy as troublemaking. Managerial training should therefore move beyond general encouragement of patient advocacy and include concrete skills for responding to team resistance, protecting advocates, and normalizing ethical voice.

Finally, hospitals should monitor advocacy climate and workplace ostracism at the unit level. Anonymous staff surveys, regular team reflection meetings, and quality-improvement reviews can be used to identify departments where nurses feel unsafe speaking up. These data should be linked to managerial accountability and targeted interventions. Without such measures, organizations may continue to endorse advocacy in principle while failing to protect the nurses who actually perform it.

### 4.6 Limitations

Several limitations should be acknowledged:

First, the cross-sectional design does not permit causal inference. Although the mediation model was specified directionally, the observed relationships should be interpreted as correlational rather than causal. Longitudinal or prospective studies are needed to clarify temporal ordering among CAB, MC, and WO.

Second, the study relied on convenience sampling from tertiary hospitals in a single Chinese city and used self-reported measures for all variables. These features may limit the generalizability of the findings and raise the possibility of common method bias, even though Harman’s single-factor test did not indicate a dominant common factor.

Third, some measures and analyses warrant further refinement. In particular, the POS scale may not have captured the forms of support most relevant to buffering advocacy-related ostracism, and the adapted moral courage measure requires further validation in Chinese nursing populations. In addition, the study focused on interpersonal outcomes and did not examine downstream consequences for patient care or organizational performance.

## 5 Conclusion

This study found that clinical advocacy behaviors were positively associated with workplace ostracism among Chinese nurses, and that moral courage partially mediated this relationship. Perceived organizational support did not significantly buffer the association. These findings contribute to nursing ethics and organizational behavior research by showing that patient advocacy, although ethically essential, may carry hidden interpersonal costs when enacted in hierarchical and high-pressure clinical environments. The study also extends Conservation of Resources theory and Social Identity Theory to the nursing advocacy context by demonstrating how ethically motivated behavior may threaten relational resources and be interpreted as deviation from team norms. Practically, hospitals should move beyond general encouragement of advocacy and implement protected escalation pathways, structured speak-up routines, junior nurse sponsorship, and managerial training to detect and prevent subtle ostracism. Future studies should use longitudinal, multisite, and multilevel designs to examine causal ordering, team-level mechanisms, and the effectiveness of interventions designed to protect nurses who advocate for patients.

## Data Availability

The datasets presented in this study can be found in online repositories. The names of the repository/repositories and accession number(s) can be found in the article/supplementary material.
